# Reactive oxygen species from non-thermal gas plasma (CAP): implication for targeting cancer stem cells

**DOI:** 10.1186/s12935-024-03523-x

**Published:** 2024-10-22

**Authors:** Amirhesam Babajani, Afshin Eftekharinasab, Sander Bekeschus, Hassan Mehdian, Faezeh Vakhshiteh, Zahra Madjd

**Affiliations:** 1 Oncopathology Research Center, Iran University of Medical Sciences (IUMS), Tehran, Iran; 2 Plasma Medicine Group, Plasma Research Institute, Kharazmi University, Tehran, Iran; 3 ZIK Plasmatis, Leibniz Institute for Plasma Science and Technology (INP), Felix-Hausdorff-Str. 2, 17489 Greifswald, Germany; 4 Department of Molecular Medicine, Faculty of Advanced Technologies in Medicine, Iran University of Medical Sciences (IUMS), Tehran, Iran

**Keywords:** Cold atmospheric plasma, Oxidative stress, Neoplasms, Reactive oxygen species, Tumor microenvironment

## Abstract

Cancer remains a major global health challenge, with the persistence of cancer stem cells (CSCs) contributing to treatment resistance and relapse. Despite advancements in cancer therapy, targeting CSCs presents a significant hurdle. Non-thermal gas plasma, also known as CAP, represents an innovative cancer treatment. It has recently gained attention for its often found to be selective, immunogenic, and potent anti-cancer properties. CAP is composed of a collection of transient, high-energy, and physically and chemically active entities, such as reactive oxygen species (ROS). It is acknowledged that the latter are responsible for a major portion of biomedical CAP effects. The dynamic interplay of CAP-derived ROS and other components contributes to the unique and versatile properties of CAP, enabling it to interact with biological systems and elicit various therapeutic effects, including its potential in cancer treatment. While CAP has shown promise in various cancer types, its application against CSCs is relatively unexplored. This review assesses the potential of CAP as a therapeutic strategy for targeting CSCs, focusing on its ability to regulate cellular states and achieve redox homeostasis. This is done by providing an overview of CSC characteristics and demonstrating recent findings on CAP’s efficacy in targeting these cells. By contributing insights into the unique attributes of CSCs and the potential of CAP, this work contributes to an advanced understanding of innovative oncology strategies.

## Introduction

Cancer remains a predominant global cause of mortality, despite substantial strides in treatment. While conventional therapies can effectively target the majority of rapidly dividing tumor cells, a subset known as cancer stem cells (CSCs) persist, contributing to cancer recurrence [1]. CSCs represent a small yet potent fraction within tumors, capable of self-renewal, differentiation, and generating diverse cell types constituting the cancerous tissue [2, 3]. Their pivotal role spans across tumor initiation, progression, resistance to treatment, and the cyclical nature of remission and relapse [4]. The resilience of CSCs presents a formidable hurdle in cancer management, as they exhibit resistance to treatments that typically eliminate the bulk of cancerous cells [5]. Hence, the imperative lies in eradicating CSCs to attain complete remission and forestall disease relapse.

In recent times, the burgeoning field of cancer research has turned its gaze towards non-thermal gas plasma, also known as low-temperature plasma (LTP), medical gas plasma technology, cold physical plasma, or cold atmospheric pressure plasma (CAP). This innovative approach harnesses the power of cold plasma technology, which creates a potent mix of electrically charged particles, reactive species, electric fields, and photons aimed at eradicating cancer cells [6]. CAP, born from electrical discharge in gases, triggers the simultaneous generation of a diverse array of reactive oxygen species (ROS) and reactive nitrogen species (RNS), collectively termed as reactive oxygen and nitrogen species (RONS) [7]. CAP embodies a dynamic amalgam of transient, high-energy entities, including electrons, ions, radicals, and excited metastable species, all physically and chemically active [8]. This plasma state is distinguished by its radiation presence, fluidic gas flow, and electric fields. Upon application to biological systems, CAP yields a profusion of ROS in the gaseous phase, comprising a spectrum of species such as hydroxyl radicals (OH⋅), superoxide radicals (O2⋅−), ozone (O_3_), atomic oxygen (O), and hydrogen peroxide (H_2_O_2_). Furthermore, it engenders various RNS, including peroxynitrite (ONOO−), nitrogen dioxide radical (NO_2_⋅), and nitric oxide (NO) [9]. The induced RONS, coupled with the oxidative stress response they elicit, have been empirically linked to anti-tumor activity, with confirmation from several scientific investigations. The initial studies of utilizing plasma in sophisticated cancer research, i.e., showing efficacy against tumors growing in vivo, were published in the early 2010s [10–12]. Subsequently, other reports demonstrated in vivo evidence of the anti-cancer capacity of plasma in several cancers including skin tumor [13–19], breast cancer [20–23], colorectal cancer [24–27], pancreatic cancer [28–31], and head and neck cancer [32].

As an innovative approach in cancer treatment, CAP emerges as a potential contender for tackling CSCs. Present therapeutic methods targeting CSCs predominantly linger in preclinical phases, primarily focusing on specific pathway interventions. Unlike conventional therapies, CAP orchestrates cellular states by modulating signaling networks to achieve redox equilibrium in a systematic manner [33]. Irrespective of distinct pathways, both cancer cells and CSCs are expected to display heightened susceptibility to CAP exposure. These attributes position CAP as a groundbreaking and promising avenue for CSC targeting. However, it’s imperative to acknowledge the limited scope of studies investigating this approach. Hence, this review aims to bridge this gap by offering a comprehensive insight into CSCs' unique traits and delving into recent findings regarding CAP’s role as a therapeutic platform against these cells. By illuminating CSC characteristics and ongoing research endeavors, this study endeavors to enrich the realm of knowledge in the pursuit of innovative and efficacious cancer treatment strategies.

## CSCs as a potential therapeutic target

According to the CSC theory, a limited population of cells, namely CSCs, accumulates genetic alterations that play a role in cancer initiation and progression by self-renewal and differentiation potential. Current cancer stemness therapeutic modalities, including targeted therapies and inhibition of stemness signaling pathways, are mainly preclinical [34]. To evaluate novel therapeutic options for CSCs, understanding CSC features such as signaling pathways, various biomarkers, and their association is necessary.

Numerous signaling pathways that play pivotal roles in governing the survival, proliferation, self-renewal, and differentiation of normal stem cells are dysregulated in the context of CSCs [35]. These signaling pathways do not operate in isolation but rather constitute an intricate web of interconnected signaling mediators that collectively oversee the growth of CSCs. Aberrant functions of signaling pathways, such as Wnt/β-catenin, JAK/STAT, PI3K/Akt/mTOR, Notch, Hedgehog, and NF-κB, have been reported in previous studies [35–39]. The complicated signal transduction pathways are far from linear. In certain instances, there is dynamic interplay and cross-communication between different pathways, orchestrating the regulation of CSCs [40]. This cooperation among CSC signaling pathways leads to the survival, self-renewal, and metastasis of CSCs [41]. Therefore, targeting multiple pathways in CSC may favor overcoming the current drawbacks of cancer therapies.

Another aspect of CSC-dependent therapeutic modalities is CSC biomarkers [42]. CSC biomarkers often intersect key cellular signaling pathways, orchestrating critical processes that sustain tumor growth and therapy evasion [43]. These biomarkers can be categorized into three main groups, including cell surface molecules, pluripotency transcription factors, and non-transcriptional enzymes.

Several surface markers associated with CSCs have been identified, although specific markers remain controversial and require additional research [44, 45]. CSC surface markers play a pivotal role in arranging signaling pathways that govern tumorigenic characteristics, cell adhesion processes, and the expression of transporter molecules such as ABCs. Among these, CD44, CD24, Prominin-1 (CD133), and Activated Leukocyte Cell Adhesion Molecule or CD166 (ALCAM) are highly significant receptors [46–50].

Pluripotent transcription factors are critical players in marking CSCs and are instrumental in maintaining the stem-like properties of these cells. Transcription factors, such as Oct4, Sox2, Nanog, and Klf4, play pivotal roles in regulating self-renewal and pluripotency in embryonic stem cells. In the context of CSCs, their expression is often associated with their ability to perpetuate themselves and give rise to several cell types within the tumor, contributing to cancer growth and resistance to treatment [51]. Unlike transcription factors or surface markers, non-transcriptional enzymes are proteins that are not directly involved in gene expression but are involved in various biochemical processes within cells. They can serve as valuable biomarkers for CSCs owing to their specific functions and roles in the biological characteristics of these cells. The most well-known non-transcriptional enzymes, such as ATP-binding cassette (ABC) and aldehyde dehydrogenase (ALDH), are upregulated in CSCs [52–55].

**Fig. 1:**
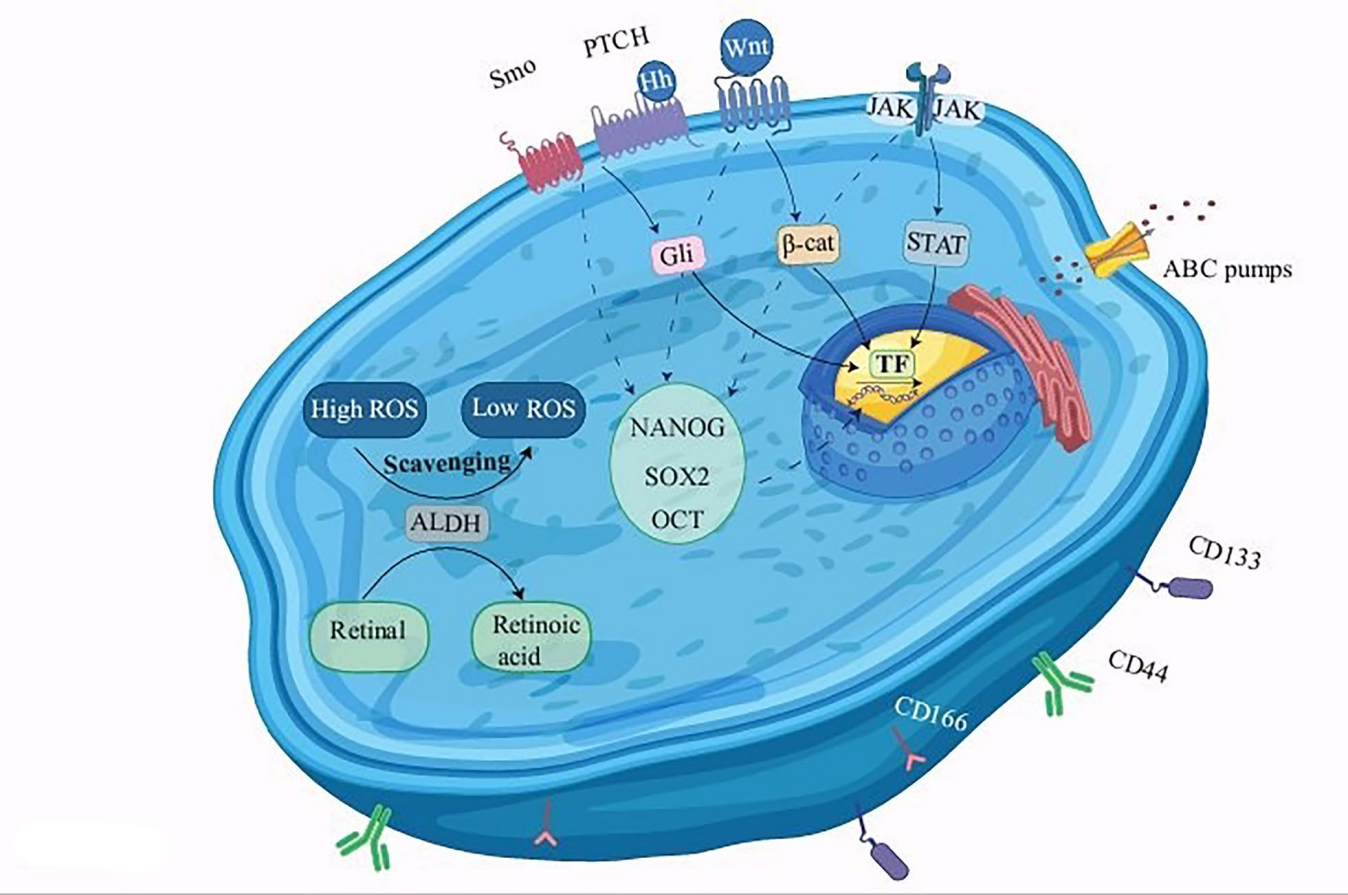
Diverse Spectrum of cancer stem cell markers. This figure depicts three prominent categories of CSC markers: surface markers, pluripotent transcriptional factors, and non-transcriptional enzymes. Surface markers such as CD166, CD133, and CD44 aid in cell identification and isolation. Transcriptional factors such as Wnt, govern pluripotency by regulating gene expression in CSCs. Non-transcriptional enzymes including ABC pumps and ALDH contribute to the intricate regulatory network. ABC pumps participate in outpouring waste molecules from cells and ALDH conducts enzymatic activity by scavenging ROS and converting Retinal to Retinoic acid which participated. Collectively, the activity of these markers offers a comprehensive insight into the characterization of stem cell populations and inducing stemness, survival, cell growth, and EMT in CSCs

ALDH is a remarkably robust CSC marker, extending its significance across a broad spectrum of cancer types. What sets ALDH apart is not just its marker status but its potential functional significance in the preservation of CSC properties. This dual role makes ALDH an appealing and promising target for eliminating CSCs [56]. The ALDH superfamily comprises 19 members, each of which is pivotal in regulating essential functions such as chemoresistance in normal stem cells and CSCs. The resistance to chemotherapy arises from converting aldehydes into less potent carboxylic acids, thereby diminishing the likelihood of toxic aldehyde accumulation within CSCs. This conversion process is facilitated by the activity of ALDHs within CSCs. Notably, high ALDH activity distinguishes CSCs, setting them apart from normal stem cells [57, 58]. Beyond their primary function in detoxifying aldehydes, ALDHs demonstrate a diverse array of capabilities. They possess the ability to directly absorb ultraviolet light, scavenge hydroxyl radicals through cysteine and methionine sulfhydryl groups, act as binding proteins for various molecules like androgen and cholesterol, and play crucial roles in antioxidation by generating NAD(P)H [59–61]. CSCs characterized by elevated ALDH expression exhibit diminished levels of ROS compared to their differentiated counterparts. This phenomenon is attributed to the upregulation of NRF2-mediated expression of essential antioxidant enzymes, including GPX3, SOD-2, and HO-1, within the ALDH-overexpressing CSC population [59, 60]. Therefore, ROS regulation in CSCs may be an avenue to target these subpopulations. Different types of CSC markers are shown in Fig. 1.

## The regulation of ROS and redox balance in CSCs

ROS collectively denote oxygen molecules exhibiting higher reactivity than free oxygen. ROS include species such as superoxide (O2−), hydrogen peroxide (H_2_O_2_), and the hydroxyl free radical (HO⋅), formed when oxygen atoms capture electrons. Nitric oxide is also broadly considered a part of ROS. In a broader context, low-to-moderate levels of ROS are pivotal for cellular proliferation, differentiation, and survival. Typically, healthy cells maintain intracellular ROS levels within a non-toxic range by delicately balancing ROS creation and removal. However, sustained elevation of ROS levels generated endogenously can induce adaptive changes that significantly contribute to tumor development, metastasis, and drug resistance across various cancer cell types [62–64]. Therefore, understanding ROS production and elimination systems is an essential step toward targeting CSCs.

### ROS production/elimination in cancer cells

Cancer cells demonstrate an elevated production of reactive oxygen species (ROS) compared to their normal counterparts [65]. This surge in intracellular ROS levels within cancer cells can stem from diverse mechanisms, including the intrinsic activation of oncogenes, suppression of tumor suppressor genes, heightened cellular metabolism, and mitochondrial dysfunction. External factors contributing to heightened ROS levels may involve abnormalities within the surrounding microenvironment and the impact of therapeutic agents [66]. ROS predominantly emanate from two principal sources: mitochondria and membrane-bound NADPH oxidases (NOXs) [67]. (i) In the course of routine cellular respiration, electrons traverse a sequence of mitochondrial complexes until they ultimately reach the conclusive electron receptor, molecular oxygen (O_2_). This sequence of events carries the potential for electron leakage from the electron transport chain, resulting in the production of O_2_−. Notably, within the mitochondria, O_2_^−^ is produced at ten specific sites. The O_2_^−^ specifically generated by mitochondrial complexes I, II, and III has been recognized for its involvement in redox signaling [68]. (ii) NOX enzymes generate O_2_^−^ through the utilization of O_2_ and NADPH. While primarily situated on the cell membrane, these enzymes can also be found on other cellular membranes [69]. Cells employ a diverse array of antioxidant mechanisms, utilizing various systems. These include small molecules such as glutathione (GSH) and reduced nicotinamide adenine dinucleotide phosphate (NADPH), as well as enzymes specialized in scavenging reactive oxygen species (ROS), including superoxide dismutase (SOD), peroxiredoxin, catalase, thioredoxin reductase, and glutathione reductase [70–73]. For instance, within mitochondria, Complexes I, II, and III release O_2_ into the mitochondrial matrix, swiftly converting it to H_2_O_2_ with the assistance of SOD_2_. Additionally, Complex III can release O_2_− into the intermembrane space, allowing its passage through voltage-dependent anion channels into the cytosol. In the cytosol, SOD1 catalyzes the conversion of O_2_− into H_2_O_2_. Furthermore, SOD1 effectively detoxifies O_2_− within the mitochondrial intermembrane space, generating freely diffusible H_2_O_2_ [68].

### ROS balance in cancer cells and CSCs

The redox status of CSCs remains uncertain; however, specific subsets of CSCs within human and mouse breast tumors have been identified with lower ROS levels than their non-tumorigenic counterparts [74]. In response to an escalating scientific demand, there is an imperative need to discern the pivotal molecular mechanisms dictating the redox equilibrium in CSCs. This exploration holds the potential to disrupt the survival mechanisms entrenched in these cells, paving the way for the eradication of cancer at its core. Studies suggest that CSCs have evolved adaptive strategies to contend with persistently heightened levels of ROS by (i) activating redox-sensitive transcription factors, which in turn enhance the synthesis of ROS-neutralizing enzymes like SODs and glutathione synthase, (ii) undergoing metabolic reprogramming, and (iii) engaging in loop effects within cellular signaling pathways [75, 76].

As an example of the enhanced scavenging system, it has been shown that interaction between a CD44 variant known as CD44v and xCT, a transporter responsible for glutamate-cystine transport, ultimately regulates the intracellular concentration of reduced glutathione. In human gastrointestinal CSCs characterized by elevated CD44 expression, an increased capability for GSH synthesis reinforces their defense against ROS [77]. Besides, CD44^+^/CD24^−^ breast CSCs exhibit reduced levels of ROS when compared to their non-tumorigenic cell counterparts. The decreased ROS levels within CSCs are correlated with an upregulation of free radical scavenging systems. When ROS scavengers in CSCs are pharmacologically depleted, their clonogenic potential significantly diminishes [78].

As the metabolic reprogramming function of CSCs, it has been demonstrated that the absence of fructose-1,6-bisphosphatase 1 (FBP1), a regulatory enzyme involved in gluconeogenesis, not only hampers oxygen consumption and the generation of ROS by downregulating the activity of mitochondrial complex I, but also leads to a metabolic shift, resulting in heightened CSC-like traits and tumorigenic potential [79]. Studies also suggest that the epigenetic regulation of metabolism may also play an essential role in the regulation of ROS in CSCs. Epigenetic mechanisms that lead to the downregulation of FBP1 enhance glycolytic activity while concurrently reducing ROS levels in basal-like breast cancer. This culminates in the activation of β-catenin signaling, contributing to the maintenance of CSCs [80]. In addition to reduced intracellular ROS in CSCs, these cells can reduce ROS in TME. It has been demonstrated that elevated CD13 expression, a CSC-related marker, can diminish ROS levels, thereby enhancing the viability of liver CSCs. Furthermore, CD13 is linked to an increased capacity for ROS scavenging in human liver CSCs [81].

**Fig. 2:**
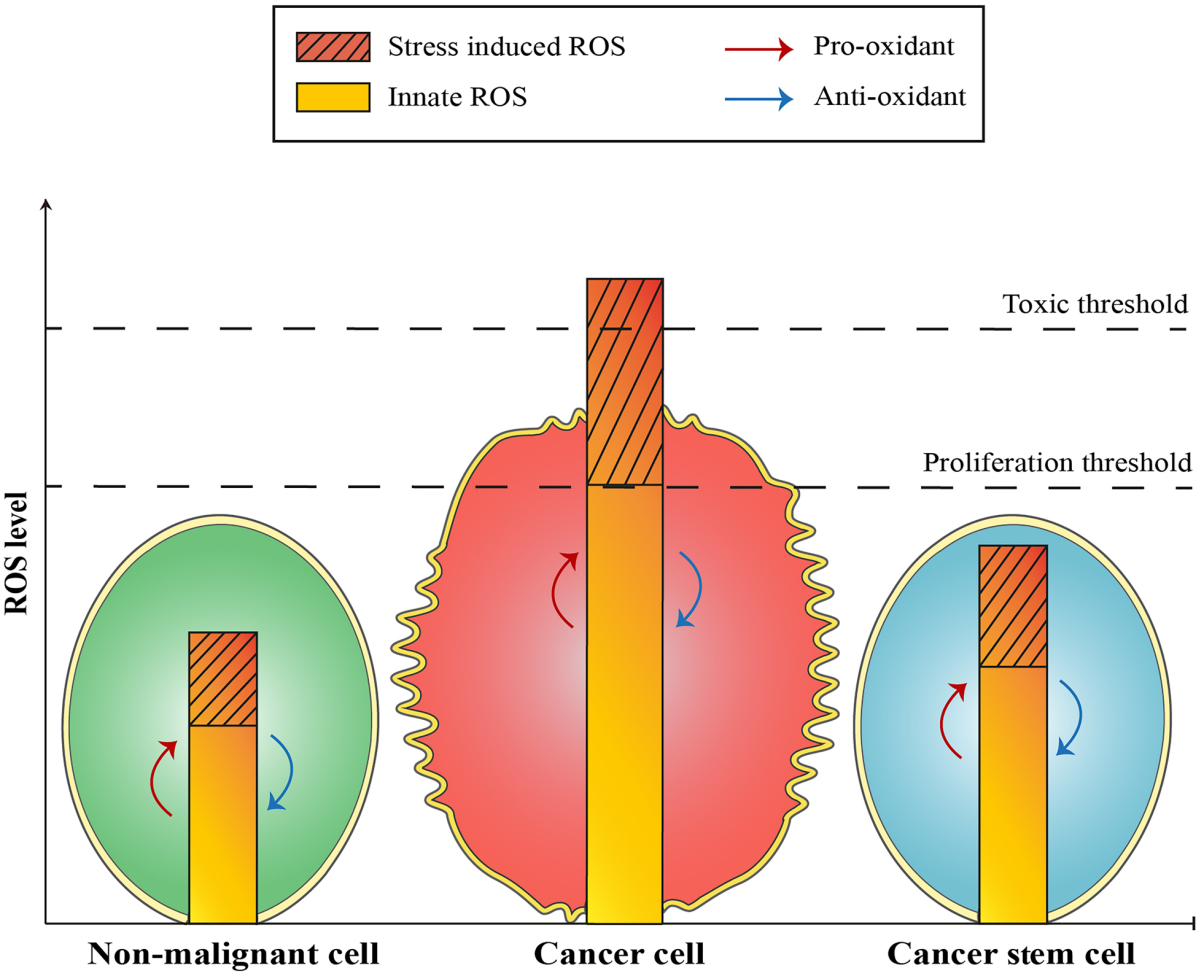
ROS Balance in Different Cell Types. Normal stem cells and CSCs exhibit lower levels of ROS attributed to robust scavenging systems. In contrast, cancer cells manifest elevated ROS levels due to dysregulated scavenging mechanisms. Upon exposure to exogenous ROS, all cells initially exhibit comparable levels; however, over time, they differentially eliminate ROS through scavenging systems. As a result, CSCs demonstrate resistance to chemotherapy and radiotherapy, while cancer cells remain susceptible to these therapeutic interventions

As an instance of the loop effects of ROS in CSCs, low ROS levels govern extracellular-signal-regulated kinase (ERK) and cyclooxygenase-2 (COX-2), leading to the expansion of the CSC population [82, 83]. Notably, there exists a negative feedback interplay between ROS and COX-2 in CSCs. ROS prompts the induction of COX-2, whereas COX-2 reduces ROS levels, potentially reinforcing CSC enrichment [83, 84]. CSCs exhibit a proficient oxidant/antioxidant machinery, acquiring a highly adaptable redox system to accommodate the local environment and withstand oxidative stress induced by heightened ROS levels resulting from conventional cancer therapies [85]. The regulation and ROS balance in normal cells, cancer cells, and CSCs are shown in Fig. 2.

### ROS as therapeutic targets in CSCs

ROS have been recognized not only as contributors to genetic instability but also as significant signaling molecules that drive various aspects of cancer, including cell proliferation, survival, angiogenesis, and metastasis [86]. CSCs operate within a finely tuned balance of ROS. This balance is carefully maintained through the interplay of ROS-producing and eliminating systems, which are integral to CSC biology. So, there are crucial challenges in recruiting ROS as therapeutic targets. In this regard, both low and high levels of ROS have the potential to prompt therapy failure in CSCs. Inducing low amounts of ROS in CSCs will enhance mentioned ROS-eliminating systems, resulting in CSC proliferation and therapeutic insufficiency [78, 87, 88]. On the other hand, a slight increase in ROS production results in maintaining stemness features and increased therapeutic resistance of CSCs via inducing epithelial-mesenchymal transition (EMT), drug resistance, and metabolic reprogramming [89–91]. Hence, there is a requisite for precise levels of ROS within CSCs to induce cell death and suppress cancer. This ROS concentration should be sufficiently high to concurrently target (i) the critical redox regulatory mechanisms governing ROS levels, (ii) essential survival factors of CSCs, and (iii) the activity of redox-sensitive survival proteins. Excessive intracellular ROS elevation can deplete and impair the antioxidant system in CSCs. The diminished ratios of GSH/GSSG and NADPH/NADP+ are indicative of ROS overproduction in cancer cells [92]. Studies have demonstrated that ROS overload induces DNA damage and sensitizes cells to the therapeutic effects of radiation therapy [89]. Additionally, surplus ROS can oxidize amino acid residues, cleave peptide bonds, and disrupt the aggregation of proteins involved in ROS scavenging [93]. Therefore, imposing both adequate and overloaded ROS levels on CSCs presents a judicious approach to cancer treatment. Furthermore, apart from ROS, reactive nitrogen species (RNS) such as nitric oxide (.NO) have also been shown to perturb cancer RNS homeostasis [94].

## Cold plasma technology in oncotherapy

The oncological research community is dedicating substantial efforts to discover new, more effective alternatives aimed at minimizing the adverse effects associated with standard cancer treatments. Among these alternatives is CAP, which shows promise in eradicating cancer cells. Similar to other conventional local ROS-generating anticancer therapies, such as radiotherapy and photodynamic therapy, CAP is administered locally and can influence multiple signaling pathways within cancer cells, facilitating their elimination [95]. Plasma, often dubbed the fourth state of matter, represents a neutral ionized gas comprised of positively charged ions, electrons, and neutral particles [96]. Besides its natural occurrence, plasma is synthetically produced by inducing ionization in a gas through electrical discharge [97]. Two primary categories of CAP devices have been identified: dielectric barrier discharge devices (DBDs) and plasma jets, extensively employed in the realm of plasma medicine [98, 99]. DBDs are powered by both KHz AC and pulsed DC sources. While DBDs predominantly utilize ambient air as the working gas for CAP generation, the discharge gap ranges from 0.1 mm to several centimeters albeit this narrow gap imposes limitations on treating larger objects [100–102]. To surmount the constraints of DBDs, plasmas ought to be generated in an unconfined space rather than within a restricted discharge gap, a characteristic exemplified in plasma jets [103]. Plasma jets offer the advantage of being applicable for direct therapy without constraints on the size of treated objects, which is paramount in medical contexts. These jets possess a cylindrical configuration with electrodes of diverse geometries, generating a high-velocity plasma stream that directly targets the desired site [103]. Plasma jets mainly use helium and argon as working gas to generate CAP for plasma medicine [104]. It should be noted that adding other gases (e.g., N_2_, O_2_, and H_2_O) to the working gas can change the chemistry of the plasma [105]. Plasma jets are powered by KHz AC, pulsed DC, microwave, and radio frequency (RF) sources [106]. In plasma medicine, plasma jets are one dominant source type investigated for biomedical purposes [107]. CAP is a shower of bullets containing RONS, electrical currents and fields, charged species (electrons and ions), and ultraviolet radiation (V)UV, which make the CAP a multi-parametric therapeutic approach [108]. The efficacy of CAP may be subject to various influencing factors, encompassing discharge type (DBD, plasma jet), parameters of the power supply (such as voltage and frequency), input power, gas composition, gas flow rate, device geometry, distance from the nozzle, and duration of treatment [108].

Gas composition is a primary and critical feature of CAP setting in cancer treatment mainly affecting ROS generation ability. Studies have used helium, air, argon, and nitrogen to target cancer cell in in vitro and in vivo studies [109–112]. Notably, some studies have used combination if these gases with oxygen and also with each other [14, 113]. Studies have demonstrated that the apoptosis rate of human breast cancer cells is higher with helium, and further increases with the addition of oxygen. Helium also generates more ROS compared to argon or nitrogen [114, 115]. However, it remains uncertain which type of gas composition is most effective for anticancer applications. Additional studies are required to identify the most efficient plasma type for each specific type of cancer.

Flow rate is another parameter of CAP device that influence ROS transportation, surface temperature, and penetration. Studies have used varying amounts of flow rates from 0.1 to 10 L/min [116, 117]. Since that there is a liquid layer between the jet and target cells, it is important to evaluate the transmission of ROS to target cells considering the flow rate of plasma jets. It has been shown that with lower gas flow rates, ROS are mainly carried by the plasma-induced linear flow deep into the liquid and spread radially at the bottom. Conversely, with higher gas flow rates, ROS are primarily transported by the supplied gas’s vortex flow, spreading radially and forming doughnut-shaped patterns at the liquid bottom. Thus, the delivery of ROS through a liquid layer to a target is significantly influenced by the balance between these two flows: the vortex flow and the linear flow [118]. The safety and temperature of a helium plasma jet on mouse skin was studied at various flow rates (1–5 L/min). The results showed both immediate and delayed skin damage, which worsened with higher flow rates. Increased flow rates raised surface temperatures to as high as 96 °C and elevated gaseous RONS concentrations [119].

The effect of frequency on CAP jets for cancer cell activity is an area of active research, as the frequency can influence the physical and chemical properties of the plasma, thereby affecting its biological interactions. The frequency of the applied voltage affects the electron density and temperature within the plasma. Higher frequencies generally increase the electron density, which can enhance the production of reactive species [120]. A study evaluated the responses of two leukemic cell lines—Jurkat T lymphocytes and THP-1 monocytes—to non-thermal plasma with varying frequencies (30, 45, 60, 75, 90, or 105 Hz). A direct relationship between frequency and cancer cell cytotoxicity and mitochondrial superoxide was observed only in Jurkat cells [121].

Treatment duration matters significantly when using CAP for cancer therapy. Longer treatment times or sooner treatment with preconditioned medium generally lead to more cell damage, effectively killing more cancer cells. A study found a clear connection between the amount of DNA damage in multicellular tumor spheroids and treatment time or the time after plasma-activated medium was first exposed to plasma. It indicated that the effectiveness of CAP on spheroids depends significantly on both the duration of exposure to plasma and the time elapsed after exposure [122]. On the other hand, extending the treatment time too much can harm healthy tissue. In a study, a direct relationship between the extent of direct skin damage and longer treatment times has been shown. The most severe damage occurred with the longest plasma treatment of 4 min, leading to significant skin burns. In contrast, indirect skin damage did not show a dependence on treatment duration. These effects were observed within 24 to 48 h after treatment, with the affected area varying unpredictably with different treatment times [119]. Therefore, finding the right treatment time is essential to balance efficacy and safety, ensuring optimal therapeutic outcomes while minimizing collateral damage induced by temperature and ROS.

**Fig. 3:**
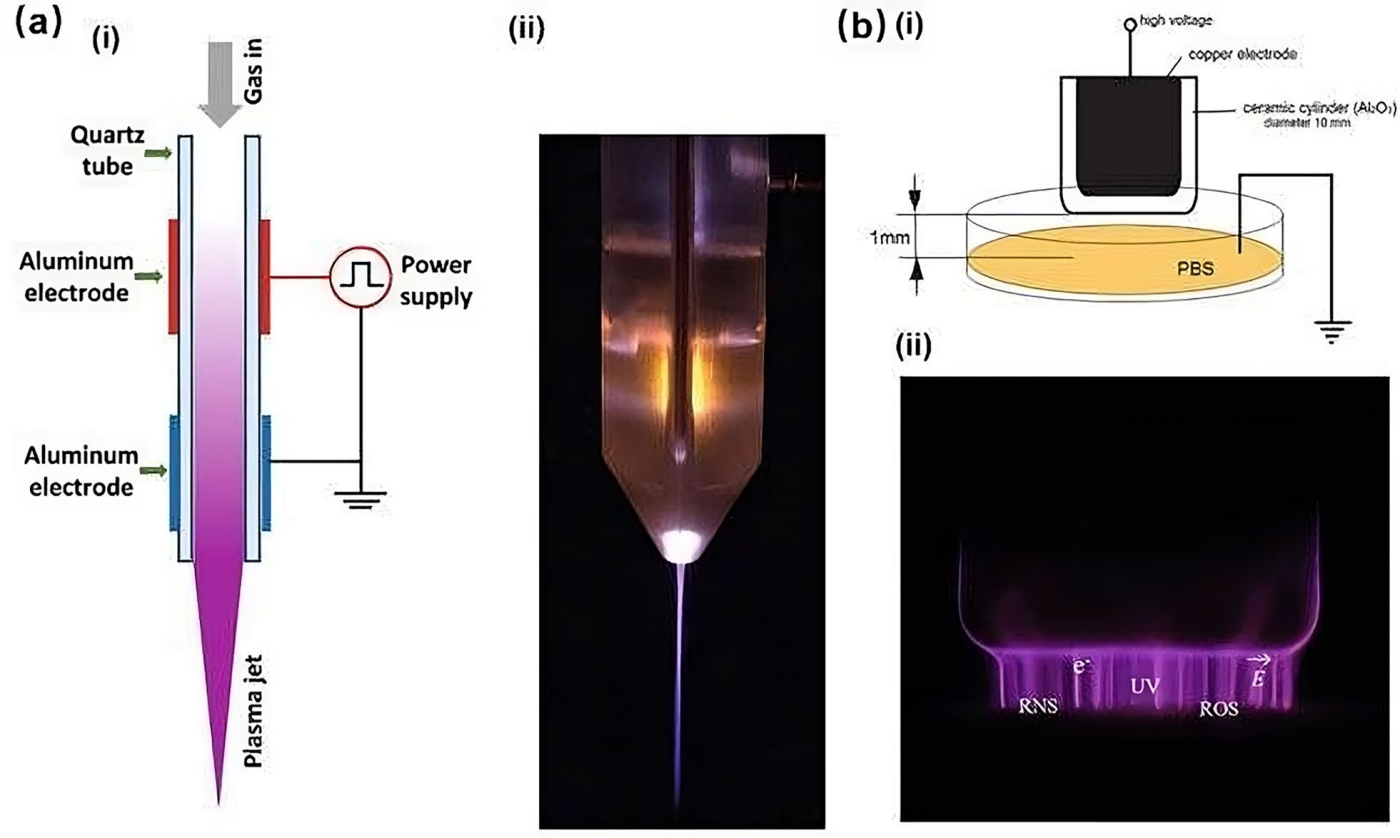
Two basic gas plasma sources utilized in medical applications. **a** Diagram depicting the plasma jet (i) and a lateral view of the jet device (ii); **b** diagram illustrating the structure of the dielectric barrier discharge (DBD) (i) and a lateral view of the plasma microfilaments generated by the DBD (ii). Reprinted with permission from [184] (under the Creative Commons Attribution (CC-BY) license provided by Springer Nature publisher) and [185] (under license number 5832520668318 published by John Wiley and Sons) with some modifications

Reactive Oxygen and Nitrogen Species (RONS) are profoundly reactive molecules generated by CAP discharges, either within the plasma itself or through interactions between the plasma and the ambient air or liquid environment [123]. Among the fleet of short-lived ROS and Reactive Nitrogen Species (RNS) engendered by CAP irradiation are hydroxyl radical (.OH), atomic oxygen (O(0)), singlet oxygen ((1)O_2_), superoxide radical anion (⋅O_2_–), ozone (O_3_), peroxynitrite (ONNO−), and nitric oxide (⋅NO) [93, 124]. Long-lived ROS and RNS species include H_2_O_2_, nitrite (NO_2_^−^), and nitrate (NO_3_^−^), respectively. In some instances, hypochlorous acid (HOCl) is also produced at significant concentrations [125, 126]. Short-lived RONS have a very short lifetime, typically ranging from microseconds to a few seconds, while long-lived species can have a lifetime of days to months or even years [127]. A schematic structure of two basic gas plasma sources utilized in medical applications is shown in Fig. 3.

Recent research has demonstrated the effectiveness of CAP in eliminating human cancer cells both in laboratory settings (in vitro) and within living organisms (in vivo) [128]. Encouraging outcomes have also emerged from specific clinical trials targeting tumors in the head and neck region [129]. The primary mechanism through which CAP potentially combats cancer is by generating Reactive Oxygen and Nitrogen Species (RONS) within the plasma, inducing oxidative stress within cancerous cells [7]. Consequently, this oxidative stress can activate signaling pathways and potentially enhance the body's anti-tumor immune response [17, 130, 131]. Yet, the precise mechanism through which CAP induces cancer cell death is not fully understood. This uncertainty likely arises from the non-specific nature of RONS as they are not targeted therapies. However, the precise mechanism by which CAP induces cancer cell death remains incompletely understood. This uncertainty likely stems from the non-specific nature of RONS, which do not selectively target cancer cells. Furthermore, each type of reactive species has the potential to affect multiple cellular signaling pathways as secondary messengers, complicating the comprehension of CAP’s cytotoxic effects [132]. Additionally, the concentration of Reactive ROS is influenced by various factors, including the specific type of CAP device used, the duration of treatment, the characteristics of the cell surface, and the biochemical composition of the sample [133, 134].

Apart from directly exposing cells and liquids to plasma discharges, significant research efforts have been directed towards investigating the therapeutic effects of liquids that have been exposed to plasma prior to their administration to cells and tissues [135]. This process is referred to as plasma-conditioned liquid (PCL), plasma-treated liquid (PTL), or plasma-activated medium (PAM) [136]. In this method, living tissue or cultured cells are solely exposed to Reactive Oxygen and Nitrogen Species (RONS) and oxidized biomolecules generated within the PTL, while being shielded from exposure to other components of the plasma. The interplay of RONS produced through either direct or indirect CAP irradiation can lead to dysregulation across various cellular levels. However, accurately identifying all RONS present in PTLs is challenging, with the most commonly measured long-lived RONS being hydrogen peroxide (H2O2) and nitrite/nitrate (NO_2_−/NO_3_−) [137–141]. It is recognized that significantly higher levels of RONS are transferred from the plasma to the liquid, as well as from the plasma to the treated tissue (such as tumors), when the plasma directly contacts the liquid [27, 142].

CAP has demonstrated no to mild side effects in animal models and clinical trials for plasma treatment of wounds, skin, and cancer [143]. In addition, plasma has the potential to overcome drug resistance in cancer, a significant challenge for conventional cancer treatments [144], although this would only be relevant in a locally restricted manner. Controllable production of RONS is another advantage of CAP that can be adjusted by plasma parameters such as gas mixture and distance for a specific purpose [16]. Currently, a plethora of in vitro and in vivo studies have substantiated the cytotoxic effects of CAP on tumor cells [145]. Within cellular redox homeostasis, the equilibrium between pro-oxidants and antioxidants governs the levels of ROS in both normal and cancerous cells. However, cancer cells typically demonstrate heightened rates, leading to elevated baseline ROS concentrations in comparison to normal cells. [75]. The idea is that while applying CAP to both normal and cancer cells increases the concentration of ROS, predominantly the ROS level in cancer cells will be above the terminal cell death threshold, offering a dose window. Since the cell membrane is the primary site of CAP interaction with cells, identifying differences in membrane properties between normal and cancer cells may help elucidate the mechanisms underlying CAP toxicity. Aquaporins, which facilitate the transport of H2O2 into cells, could be instrumental in this process [146], are sometimes found in more significant amounts in cancer cell membranes. Therefore, it has been hypothesized that an increased expression of aquaporin in cancer cells may increase the sensitivity of these cells to CAP treatment compared to normal cells [147]. However, an experimental study investigating the sensitivity of 36 cancer cell lines to plasma treatment and, in parallel, surveying the expression of 11 aquaporin molecules in the cell membranes of each cell line did not confirm a correlation between the expression of any of the aquaporins and sensitivity to plasma treatment [148]. Cholesterol, often found in lower amounts in tumor cells than in non-malignant cells, reduces the diffusion of RONS across cell membranes, making them more susceptible to oxidative stress [149, 150]. High cholesterol levels in normal cells protect them from the penetration of RONS. Studies have shown that RNS and ozone can oxidize phospholipid bilayers and penetrate cells more efficiently than hydrophilic ROS, such as ^⋅^OH and H_2_O_2_ [151]. Therefore, a low amount of cholesterol in cancer cells causes them to be killed selectively by CAP. This hypothesis, made through modeling efforts, has been confirmed by experimental evidence in 36 cancer cell lines [148].

### Perspective of CAP in targeting CSCs

**Fig. 4:**
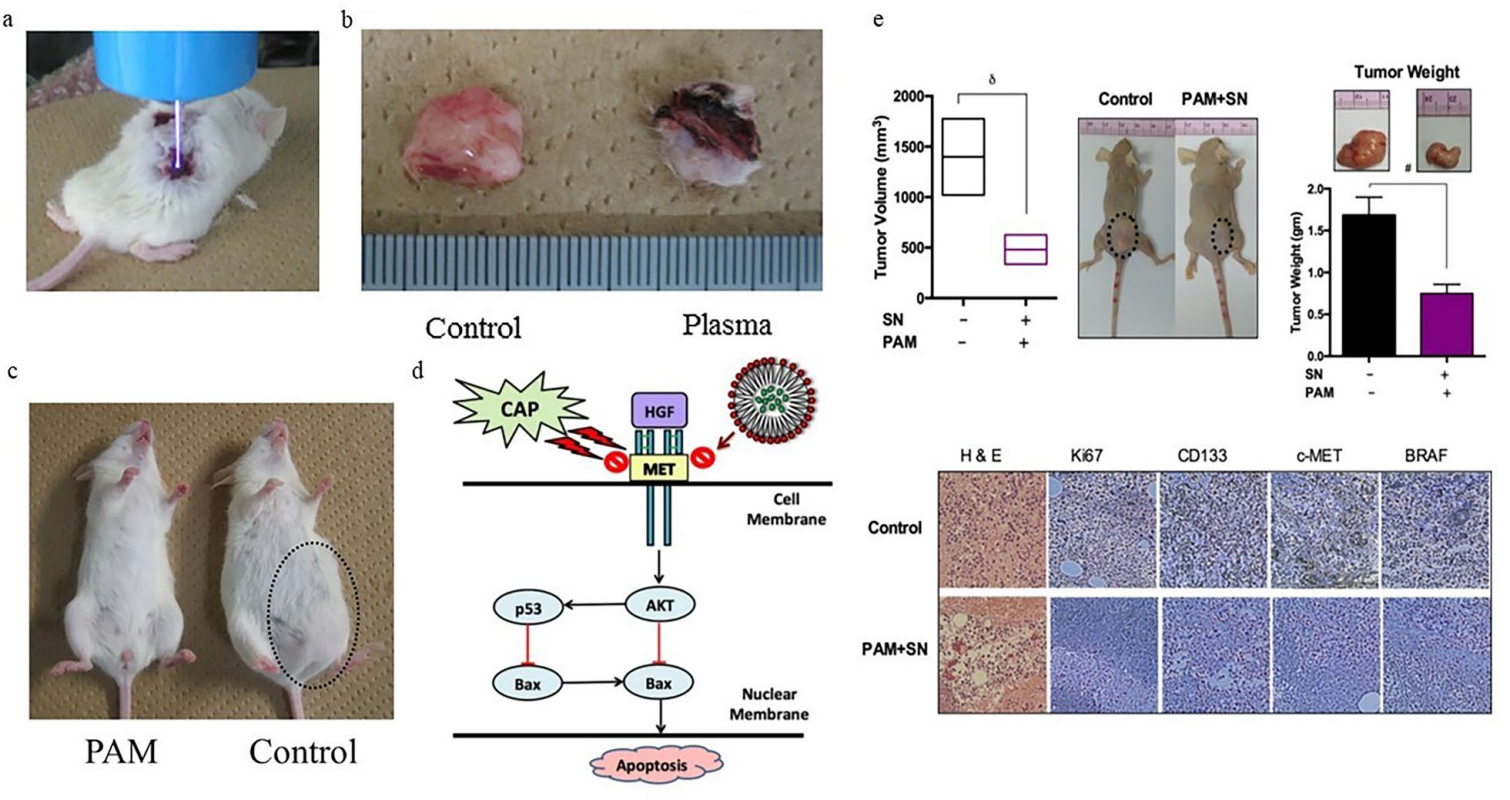
In vivo experiences of CAP to target CSCs. **a**, **b** The study explores the effects of nonequilibrium atmospheric pressure plasma (NEAPP) treatment combined with the anti-cancer drug cisplatin on human uterine endometrioid adenocarcinoma cells and tumor xenograft mice. NEAPP treatment is more effective than cisplatin alone in targeting both ALDH-low and ALDH-high cells. In tumor xenograft mice, NEAPP irradiation reduces ALDH expression in tumors (reprinted with permission from [153] under license number 5832490781169 published by John Wiley and Sons with some modifications). **c** Plasma-activated medium (PAM) selectively induces apoptosis in cancer cells without harming normal cells. PAM reduced the viability of CSC populations of endometrioid carcinoma and gastric cancer cells with high ALDH levels. The combination of PAM and cisplatin was more effective at killing CSCs than either treatment alone (reprinted with permission from [154] under license number 5832500194861 published by John Wiley and Sons with some modifications). **d** The study explored the combined treatment of CAP and silymarin nanoemulsion (SN) on human melanoma cells. Diagram showing how SN and CAP together induce apoptosis via the HGF/c-MET pathway. **e** In vivo results of CAP and SN effects on melanoma showed significant reduction in tumor weight and size. The combined treatment increased cellular toxicity in a time-dependent manner. Caspases 8, 9, 3/7, PARP-1, and apoptotic genes also increased in the dual-treated group, indicating blockage of the HGF/c-MET pathway. EMT markers (E-cadherin, YKL-40, N-cadherin, SNAI1) decreased, along with melanoma cell (BRAF, NAMPT) and stem cell (CD133, ABCB5) markers (reprinted with permission from [157] under the Creative Commons Attribution (CC-BY) license provided by BMC publisher with some modifications)

One of the primary studies investigating the impact of CAP on CSCs was conducted in 2014. Using ALDH activity as a marker for identifying CSCs, the group studied the consequence of direct CAP irradiation on human uterine endometrial adenocarcinoma CSCs. The results indicated that CAP effectively eliminated CSCs in adenocarcinoma cells [152]. Subsequently, in 2015, the same group investigated the combined effect of cisplatin and CAP on an animal model of uterine endometrioid adenocarcinoma. As per the findings of this research, the use of CAP treatment was demonstrated to be more effective than cisplatin alone in targeting CSCs (ALDH-high cells). In a xenografts mouse model, CAP induced apoptosis in the tumor cells, inhibited their proliferation, and decreased the expression of ALDH, a crucial non-transcriptional enzyme biomarker for CSCs. These findings imply that CAP may potentially diminish the stemness characteristics of CSCs within the tumor bulk [153]. In 2017, the same group explored the potential of PTL as an anti-tumor therapy targeting CSCs in endometrioid carcinoma and gastric cancer cells. They used the CAP device powered by a 60 Hz AC high-voltage supply (10 kV peak-to-peak), with argon gas flowing at a rate of 2 standard liters per minute. The finding of this study indicated that PTL effectively killed CSCs, similar to direct CAP, suggesting its potential as a new approach to target CSCs. Moreover, the combination of PTL and cisplatin appeared to be more effective in eradicating cancer cells than either PTL or cisplatin alone [154]. In 2015, Trink et al. showcased the responsiveness of two sets of CSCs, derived from a singular patient tumor and differing in aggressiveness levels, to CAP irradiation. Their study revealed that both heterogeneous CSC populations were vulnerable to plasma irradiation, albeit with varying degrees of sensitivity. Notably, the more aggressive subset of stem cells displayed heightened susceptibility to CAP irradiation when contrasted with the less aggressive counterpart [155]. One hypothesis is that the size of cells highly impacts the effects of CAP on CSCs. It has been shown that CSCs are smaller than cancer cells, so it can be concluded that the smaller the size, the better the therapeutic effects of CAP [156]. In 2019, Adhikari et al. used DBD device operated with air at a flow rate of 1.5 L/min. The applied root mean square (rms) voltage and current were 1.33 kV and 12 mA, respectively, with a frequency of 58 kHz. They demonstrated that the combined application of silymarin nanoparticles (SN) and CAP resulted in heightened cellular toxicity of melanoma CSCs over time in vitro. Furthermore, a notable increase in the production of RONS was observed in the dual-treated samples compared to the control. Additionally, the levels of Caspase 8, 9, 3/7, PARP, and apoptotic genes were elevated in the dual-treated group, while a decrease in EMT markers (E-cadherin, YKL-40, N-cadherin, and SNAI1) was noted, along with a reduction in CSC surface markers (CD133, ABCB5). These findings offer a basis for combining SN and CAP to enhance the effectiveness of therapeutic strategies for melanoma [157]. In 2019, Kaushik et al. unveiled a fresh mechanism of plasma immunomodulation that bolsters an anti-tumorigenic impact by influencing monocyte-derived macrophages. According to this research, CAP spurred the activation and transformation of monocyte cells into macrophages, as evidenced by the observed expression of various cytokine/chemokine markers. Moreover, CAP prompted a more pronounced shift towards pro-inflammatory (M1) macrophages. These activated macrophages displayed a preference for fostering anti-tumorigenic immune responses against metastasis development and the sustenance of CSCs in solid cancers in vitro. The conversion of monocytes into anticancer macrophages demonstrated a potential to enhance the efficacy of CAP treatment, particularly noteworthy in reshaping the pro-tumor inflammatory milieu, countering the influence of highly resistant immunosuppressive tumor cells often associated with the risk of tumor recurrence [158]. Another aspect of evaluating CAP-CSCs interaction is to demonstrate its role in the tumor extracellular matrix (ECM). To mimic the intricacy of the bone microenvironment, Tornín et al. [159] developed a three-dimensional (3D) model of osteosarcoma through a bone-like scaffold made of collagen type I and hydroxyapatite nanoparticles to replicate the bone microenvironment. Based on this study, the 3D environment protected cells from plasma-activated ringer saline (PAR)-induced cytotoxicity by scavenging and reducing RONS levels generated by CAP. Additionally, the 3D culture condition induced the expression of several RONS-protective genes and facilitated the survival of osteosarcoma subpopulations by enhancing the cancer stem-like features of osteosarcoma cells, promoting cell proliferation and facilitating adaptation to oxidative stress caused by PAR treatment [159]. In summary, the interaction between cancer cells and the ECM not only facilitates the conversion of tumor cells into CSCs but also acts as a mechanism for maintaining CSC niches, thereby supporting and preserving specific CSC characteristics [160]. Tornín et al. [87] examined the impact of CAP on CSC subpopulations and tumor progression in vivo. Their study revealed that low doses of Plasma-Treated Liquid (PTL) heightened pro-stemness factors and the self-renewal capacity of osteosarcoma cells, consequently enhancing in vivo tumor growth potential. However, the detrimental pro-stemness signals mediated by PTL were counteracted when combined with the STAT3 inhibitor S3I-201. These findings unveiled an unfavorable stem cell-promoting attribute of PTL in cancer and advocated for the utilization of combination therapies with STAT3 inhibitors as an effective therapeutic strategy for osteosarcoma [87]. In 2022, Aggelopoulos et al. investigated the effects of direct and indirect CAP treatment, facilitated by advantageous nanosecond pulsed discharge, on breast cancer cells with varying malignant phenotypes and estrogen receptor (ER) status. CAP treatment induced significant phenotypic alterations and apoptosis in both ER-positive and ER-negative cells. Furthermore, CAP markedly reduced CD44 expression and influenced the expression of proteases and inflammatory mediators [161]. Similarly, in 2022, Lee et al. conducted a study to assess the impact of CAP irradiation on ovarian CSCs using CAP with argon served as the feed gas beneath the dielectric cap. The gas flow rate was set at 1 L per minute, and voltage and current adjustments were made using a variable power supply. The system operated at 20 kV voltage, 8.4 mA current, and a frequency of 20 kHz. Their findings demonstrated that CSCs resistant to conventional chemotherapy exhibited sensitivity to PTL in a dose-dependent manner. PTL treatment also decreased the expression of CSC markers, sphere formation, and the population of ALDH or CD133 positive (ALDH+ or CD133+) cells. Moreover, the researchers explored the effects of combining PTL with other chemotherapeutic agents on ovarian CSCs in vitro. PTL demonstrated synergistic cytotoxicity with cisplatin but not with paclitaxel and doxorubicin. Consistent results were observed in a xenograft model of peritoneal metastasis established through intraperitoneal spheroid injection, indicating the promising potential of PTL as intraperitoneal chemotherapy to enhance anti-tumor efficacy while minimizing adverse effects [162]. Taken together, these findings indicate that CAP could potentially reprogram cisplatin-resistant ovarian cancer cells to a cisplatin-sensitive state by disrupting the antioxidant axis. It is noted that cisplatin resistance often correlates with heightened expression of antioxidant proteins [163]. Consequently, targeting the antioxidant axis of malignant cells with specific agents may represent a viable strategy for regulating cancer stemness. Furthermore, to understand the diverse therapeutic effects of CAP in combination with chemotherapeutic agents, it is necessary to gain insight into their mechanisms of action. The three mentioned chemotherapeutic drugs primarily increase ROS by impairing mitochondrial function. Therefore, CAP appears to have additional therapeutic effects on cancer cells beyond influencing ROS levels [164–166]. In 2022, Dai et al. conducted an investigation into the therapeutic potential of CAP for breast cancer utilizing a comprehensive approach involving whole transcriptome sequencing, in vitro and in vivo assays, as well as clinical samples. Their study proposed that CAP could effectively target cancer stemness by inhibiting the AQP3/FOXO1 axis. The suppression of FOXO1 phosphorylation hindered its regulatory functions in sustaining cancer stemness, including the modulation of ALDH1 and IL6. Additionally, the researchers observed heightened anti-cancer efficacy when CAP was combined with Atorvastatin both in vitro and in vivo. Overall, the study highlighted CAP as a promising oncotherapy that could be utilized independently or in conjunction with other therapeutic modalities to combat cancer [167]. In vivo experiences of using CAP for targeting CSCs is shown in Fig. 4.

On the other hand, Lv et al. aimed to establish primary organoid models and identify common inflammatory cytokines capable of targeting cancer stemness as an innovative strategy for managing colorectal cancer. Their findings revealed interferon-gamma (IFNγ) as a key cytokine capable of halting intestinal stem cells through the IFNγ/IFNGR2/APC/TCF4/GPX4 axis, thereby triggering GPX4-dependent ferroptosis and eliminating colorectal CSCs. Furthermore, they demonstrated the synergistic efficacy of indirect CAP with IFNγ in inducing colorectal cancer cell ferroptosis via the same axis, suggesting a potential innovative approach for treating colorectal cancer [168]. The comprehensive effects of cold plasma on CSCs and stemness characteristics across various cancer types have been summarized in Table 1.

**Table 1:** The effects of cold plasma on CSCs and stemness characteristics in various cancers entities

Cancer entity	Model	Responses to plasma	CAP	Treatment modality	Refs.
• Endometrioid adenocarcinoma • Gastric carcinoma	In vitro	• Inducing apoptosis • Reducing ALDH^high^ cancer cell population	Plasma Jet	Direct	[152]
Endometrioid adenocarcinoma	In vitro In vivo	• Inducing apoptosis • Reducing ALDH^high^ cancer cell population	Plasma jet	Direct	[153]
Ovarian cancer	In vitro	• Reducing CSC viability	Helium plasma jet	Direct	[155]
• Endometrioid adenocarcinoma • Gastric carcinoma	In vitro In vivo	• Increased production of ROS • Reducing cancer cell viability • Reducing ALDH^high^ cancer cell population	Argon plasma jet	Indirect (combined with cisplatin)	[154]
Melanoma	In vitro In vivo	• Increased production of ROS and RNS • Inducing apoptosis • Inducing DNA damage • Reducing EMT markers	Air DBD	Direct	[157]
Glioblastoma	In vitro* (3D)* In vivo	• Inducing differentiation of monocytes into M1 macrophages • Enhancing immune responses against cancer • Reducing EMT markers • Inhibiting cancer stem cell traits	Nitrogen DBD	Direct & indirect	[158]
Osteosarcoma	In vitro* (3D)*	• 3D environment-protected cells CAP therapeutic effects • 3D culture reduced amount of RONS generated by CAP • 3D favored the stemness phenotype of osteosarcoma cells	Argon plasma jet	Indirect	[159]
Breast cancer	In vitro	• Inducing phenotypic alteration • Inducing apoptosis in both ER^+^ and ER^−^cancer cells • Reducing CD44 protein expression	Air DBD	Direct & indirect	[161]
Ovarian cancer	In vitro In vivo	• Reducing the expression of stem cell markers and sphere formation • Reducing ALDH^high^ and CD133^+^ cancer cell population • Inducing apoptosis	Argon DBD	Indirect (combined with cisplatin)	[162]
Breast cancer	In vitro In vivo Ex vivo (clinical)	• Inhibiting FOXO1 phosphorylation • Reducing ALDH1 and IL6 • Reducing stemness • Reducing CSC viability	Helium plasma jet	Indirect (combined with Atorvastatin)	[167]
Osteosarcoma	In vitro (3D) In vivo	Using STAT3 inhibitor increased therapy outcome	Argon and Helium plasma jet	Indirect	[87]
Colorectal cancer	IN vitro	• Inducing ferroptosis	Helium plasma jet	Indirect	[168]

*ALDH* aldehyde dehydrogenase, *CAP* cold atmospheric plasma, *CSCs* cancer stem cells, *DBD* dielectric barrier discharge, *EMT* epithelial–mesenchymal transition, *ER* estrogen receptor, *FOXO1* Forkhead Box O1, *IL6* Interleukin 6, *M1* M1 macrophages, *RNS* reactive nitrogen species, *ROS* reactive oxygen species, *STAT3* signal transducer and activator of transcription 3

**Fig. 5:**
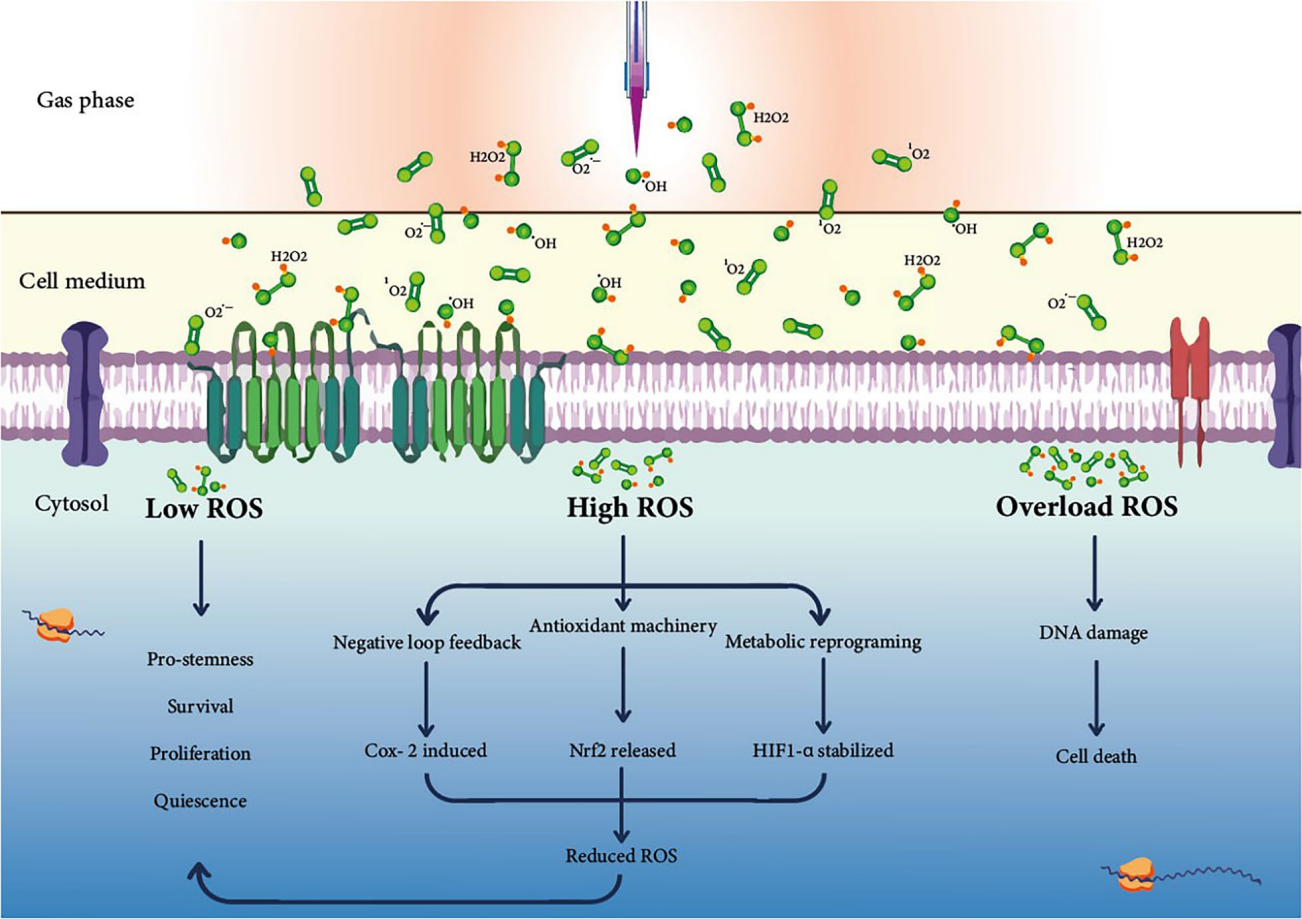
Response of CSCs to CAP treatments. CSCs exhibit different responses to three reactive oxygen species (ROS) regimes. At low ROS levels, CSCs cells promote their survival, stemness, and quiescence. When exposed to high ROS levels, they activate COX-2, antioxidant machinery, and hypoxia to maintain low ROS levels. Disrupting the redox balance in CSCs by ROS overloading can lead to their death by causing DNA damage

In summary, CAP represents a multimodal therapeutic approach aimed at suppressing and eradicating CSCs with tumorigenic capabilities. The mechanism through which CAP induces the elimination of CSCs is contingent upon the specific strategy employed, highlighting the versatility of this treatment. The ultimate therapeutic outcome is influenced by a multitude of plasma parameters, including but not limited to plasma parameters (flow rate and gas mixture), the nature of discharges (whether direct or indirect), combination with other treatment modalities, the type of cancer being targeted, and levels of ROS. The therapeutic effects of CAP on CSCs by interfering with ROS balance are shown in Fig. 5.

### ROS generation by CAP vs. light- and ultrasound-based cancer therapies

Photodynamic therapy (PDT), sonodynamic therapy (SDT), and photothermal therapy (PTT) represent innovative modalities with significant potential in cancer therapy. Each of these approaches harnesses distinct mechanisms to target and destroy cancer cells, offering unique advantages in specific clinical contexts.

Comparing CAP with PDT, SDT, and PTT reveals distinct approaches with unique mechanisms for cancer therapy. CAP operates by generating a complex mixture of ROS and RONS at room temperature. These RONS, including ozone, hydroxyl radicals, and nitric oxide, induce oxidative stress in cancer cells, disrupt cellular membranes, and alter signaling pathways, ultimately leading to cell death. CAP’s broad spectrum of RONS enables it to target various cancer types, both superficially and within deeper tissues, making it a versatile therapeutic option [169, 170].

In contrast, PDT relies on a photosensitizer activated by specific light wavelengths to produce predominantly singlet oxygen and other ROS within targeted cancer cells. This localized ROS generation is effective for treating superficial cancers and has been successful in dermatological conditions and early-stage cancers. PDT’s precision in ROS delivery allows for targeted therapy and minimal damage to surrounding healthy tissue, although its effectiveness can be limited by light penetration depth [171, 172].

SDT, using ultrasound to activate sonosensitizers and generate ROS within tumors, offers deeper tissue penetration compared to PDT. This makes SDT suitable for treating internal solid tumors such as those in the liver, pancreas, and prostate. The ROS produced in SDT induces oxidative stress and cellular damage, contributing to cancer cell death. SDT's ability to reach deeper tissues expands its therapeutic potential beyond superficial lesions [173, 174].

PTT, on the other hand, utilizes light-absorbing agents like nanoparticles to convert light energy into heat, leading to thermal ablation of cancer cells. While PTT primarily relies on photothermal effects, it can also induce mild ROS production as a secondary effect. PTT’s precise spatial and temporal control allows for targeted therapy and minimal damage to surrounding tissues, particularly beneficial for treating solid tumors in various organs [175–177].

In comparison, CAP’s mechanism of action through RONS generation offers distinct advantages, including its ability to penetrate both superficial and deep-seated tumors effectively [145]. Its non-invasive nature, broad applicability across different cancer types, and potential for combination therapies with other modalities highlight CAP as a promising frontier in cancer treatment research [178].

### Clinical application of CAP in cancer therapy

There is a limited amount of clinical literature available regarding the use of CAP in treating cancer, especially targeting CSCs. The important feature should be considered in clinical application of CAP in cancer consist of efficacy, safety, treatment parameters, combination therapy, and long-term Outcomes. In this regard, Metelmann et al. explored the use of CAP in six patients suffering from locally advanced squamous cell carcinoma (SCC) of the oropharynx, which was characterized by open infected ulcerations. Using a jet plasma device (kINPen MED), patients underwent three treatment cycles within one week, with each session lasting 1 min/cm^2^ at an 8 mm distance, followed by a week of rest. The findings revealed significant improvements, including decreased odor and reduced need for pain medication, as well as enhancements in social functioning and emotional well-being. Notably, two patients experienced partial remission lasting over nine months, supported by biopsy results showing apoptotic tumor cells and a desmoplastic reaction in the connective tissue [179]. In another study, Canady Helios Cold Plasma (CHCP), a CAP device, underwent Phase I evaluation in 20 patients with stage IV or recurrent solid tumors who underwent surgical resection followed by intra-operative CHCP treatment. The trial focused primarily on safety, assessing secondary outcomes such as non-local regional recurrence (LRR), overall survival rates, cancer cell death, and tissue preservation. Results indicated that CHCP had no detrimental effects on intraoperative physiological parameters and did not cause any adverse events. Over a 26-month follow-up period, CHCP treatment demonstrated promising overall response rates of 69% for patients undergoing complete (R0) tumor resections and 100% for those with R0 resections with microscopic positive margins (R0-MPM). Survival rates varied across patient groups. Notably, CHCP proved safe, specifically targeting cancer cells, and effectively controlled LRR, particularly in patients with complete or near-complete tumor resections (R0 and R0-MPM) [180]. The clinical studies on CAP in cancer treatment have been described in Table 2.

**Table 2:** Clinical use of cold plasma in malignant and pre-malignant pathologies

Pathology	Phase	Enrolled	Responses to plasma	CAP	Refs.
Stage IV or recurrent solid tumors	I	20	• Control of LRR in patients with R0 and R0-MPM resections	Canady Helios™ Cold Plasma System	[180]
SCC of the oropharynx	N/A	6	• Reduced odor and decreased pain medication • Improved social function • Partial remission lasting at least nine months • Reduced apoptotic tumor cell count and desmoplastic reaction	kINPen MED	[179]
Wart or molluscum lesion	III	40	No study results posted	FE-DBD	NCT05937672
Cervical intraepithelial neoplasia	N/A	63	No study results posted	Low-temperature argon plasma	NCT03218436
Warts and molluscum lesion	IV	17	No improvement	FE-DBD	NCT05070754
FAP	N/A	10	No study results posted	Low energy argon plasma coagulation	NCT06435533

*SCC* squamous cell carcinoma, *FAP* familial adenomatous polyposis

## Challenges and future directions

CSCs are a primary target in the development of novel cancer treatment strategies aimed at overcoming metastasis and recurrence and improving patient survival. CAP seems to be a potential therapeutic method for targeting CSCs locally, though the effects of CAP on CSCs are not yet well understood. CAP is a multi-parametric treatment approach that requires precise control over plasma parameters, such as flow rate and gas mixture, to achieve optimal results. The kind of applied modalities of CAP, direct vs. indirect, might also be a determinant in CAP-related results. By summarizing the available studies, we concluded that while some studies have shown that CAP can eliminate CSCs, others have reported increased CSC features following treatment with CAP [159], which may be due to sublethal doses applied. Additional studies are required to recognize the mechanisms underlying the effects of CAP on CSCs and to optimize treatment outcomes. CAP stands out from other therapeutic strategies by modulating cell states through the orchestration of signaling networks toward redox homeostasis [7]. CSCs possess a robust oxidant/antioxidant machinery, allowing them to adapt and coexist with their surrounding environment while withstanding oxidative stress caused by radiotherapy and chemotherapy. The enhanced antioxidant capability is an acknowledged characteristic contributing to reduced oxidative stress levels in CSCs in comparison to the bulk of tumor cells [162]. One promising approach could be overwhelming CSCs with oxidative stress, thereby disrupting their redox equilibrium. In targeting CSCs with CAP, it is essential to distinguish between three distinct ROS regimes that CSCs encounter: low, high, and overload. CSCs maintain low levels of ROS, which contribute to their quiescence and resistance to therapy. However, when CSCs are exposed to high levels of ROS, it induces hypoxia, which further enhances their resistance to treatment [181]. It should be noted that ROS in cancer is context-dependent, and those critical ROS doses may vary across cancer types. Therefore, a comprehensive understanding of the intricate relationship between ROS and CSCs is crucial for developing CAP strategies that can selectively eliminate CSCs. In general, it is anticipated that CAP can be employed as part of combination therapy rather than as a standalone approach in oncology [131, 182]. CAP has the potential to synergize with other anticancer drugs, thereby enhancing their effectiveness and overcoming drug resistance. This capability likely stems from CAP’s ability to counteract the mechanisms underlying the development of drug resistance in cancer cells, thereby rendering them more responsive to medication [144]. Moreover, CAP can stimulate the immune system, promoting the recognition and elimination of cancer cells. This immunostimulatory property not only enhances the efficacy of immunotherapies but also suggests the possibility of synergistic effects when combined with CAP treatment [131, 183]. As a prospective sensitizer of the TME with the capability of targeting CSCs, CAP holds promise of independently or in combination with established standard treatments to advance the effectiveness of existing anticancer drugs [144].

Despite several advantages in this field, effectively determining the optimal doses of CAP for maximum efficacy across diverse pathological conditions still poses a challenge. Additionally, the technical combination of CAP with current therapeutic approaches for clinical administration to achieve synergistic effects remains an ongoing challenge [33]. Moreover, the identification of predictive biomarkers that can accurately anticipate the sensitivity and treatment response of tumors to CAP exposure is crucial. Addressing the mentioned challenges necessitates a comprehensive understanding of the molecular mechanisms underlying CAP’s distinctive characteristics. Positively, these challenges can be addressed in the situation of many gas plasma technologies already approved as medical products, i.e., specific goods to target a medical (not cosmetic) condition, especially in Europe [143]. On this basis, the continued development of plasma therapies based on clinical experience is expected to be accelerated.

## Conclusion

CAP emerges as a novel and promising technology in the realm of cancer treatment. While it presents certain drawbacks, exploring new therapeutic targets holds the key to overcoming these limitations. CSCs, identified as the main aggressive population, stand out as a particularly promising target for intervention. To effectively counteract the challenges associated with CAP treatment, directing attention towards the exclusive balance of ROS in CSCs proves to be a significant avenue. The intricate interplay of ROS in CSCs presents an opportunity for tailored therapeutic strategies. In light of this, it becomes imperative for future studies to delve deeper into the multifaceted aspects of ROS balance within CSCs.

## Data Availability

No datasets were generated or analysed during the current study.

## Abbreviations

ABCs: ATP-Binding Cassettes
AQP3: Aquaporin 3
CAP: Cold atmospheric plasma
CD44v: CD44 variant
COX-2: Cyclooxygenase-2
CSCs: Cancer stem cells
DBDs: Dielectric barrier discharge devices
EMT: Epithelial–mesenchymal transition
ERK: Extracellular-signal-regulated kinase
FBP1: Fructose-1,6-bisphosphatase 1
FOXO1: Forkhead Box O1
GPX3: Glutathione peroxidase 3
GPX4: Glutathione peroxidase 4
GSH: Glutathione
HOCl: Hypochlorous acid
HO-1: Heme oxygenase-1
IFNγ: Interferon-gamma
IFNGR2: Interferon gamma receptor 2
LTP: Low-temperature plasma
NRF2: Nuclear factor erythroid 2-related factor 2
NO: Nitric oxide
NO2⋅: Nitrogen dioxide radical
NOXs: NADPH oxidases
O3: Ozone
O(0): Atomic oxygen
O2⋅−: Superoxide radicals
O2−: Superoxide
OH⋅: Hydroxyl radicals
ONNO−: Peroxynitrite
PAM: Plasma-activated medium
PTL: Plasma-treated liquid
RNS: Reactive nitrogen species
ROS: Reactive oxygen species
RONS: Reactive oxygen and nitrogen species
SOD: Superoxide dismutase
SOD-2: Superoxide dismutase 2
TCF4: Transcription factor 4
VUV: Vacuum ultraviolet
xCT: Cystine-glutamate transporter
